# Supplementary material for: Effect of government revenue on economic growth of sub-Saharan Africa: Does institutional quality matter?
Source: PLoS One. 2023 Nov 29;18(11):e0293847. doi: 10.1371/journal.pone.0293847 (PMC10686516; doi:10.1371/journal.pone.0293847)
Supplement: S1 Table — (DOCX) [file pone.0293847.s001.docx]

**Variables of the study and their corresponding license**

| **Variables of the study** | **License URL** |
| --- | --- |
| Tax revenue (% of GDP) | <https://datacatalog.worldbank.org/public-licenses#cc-by> |
| Population, total | <https://datacatalog.worldbank.org/public-licenses#cc-by> |
| GDP per capita growth (annual %) | <https://datacatalog.worldbank.org/public-licenses#cc-by> |
| GDP per capita (constant 2015 US$) | <https://datacatalog.worldbank.org/public-licenses#cc-by> |
| Foreign direct investment, net inflows (% of GDP) | <https://datacatalog.worldbank.org/public-licenses#cc-by> |
| Trade (% of GDP) | <https://datacatalog.worldbank.org/public-licenses#cc-by> |
| Institutional Quality Index | <https://www.heritage.org/index/download> |
