# Supplementary material for: Effect of government revenue on economic growth of sub-Saharan Africa: Does institutional quality matter?
Source: PLoS One. 2023 Nov 29;18(11):e0293847. doi: 10.1371/journal.pone.0293847 (PMC10686516; doi:10.1371/journal.pone.0293847)
Supplement: Attachment — Submitted filename: Article 6 Plos review response.docx [file pone.0293847.s002.docx]

**Date: 3^rd^ October, 2023**

**TO: PLOS ONE**

**Subject: Submission of a revised version of the manuscript PONE-D-23-27778 for evaluation**

**Title:** Effect of government revenue on economic growth of Sub-Saharan Africa: Does institutional quality matter?

**Manuscript No.: PONE-D-23-27778**

^*^[isubalewmsc@gmail.com](mailto:isubalewmsc@gmail.com)/ [isubalewd@wollegauniversity.edu.et](mailto:isubalewd@wollegauniversity.edu.et)

Dear Reviewers,

Greetings of the day!

We are thankful to the reviewers for taking the time to assess our manuscript, for their careful reading, and for their suggestions and valuable comments which helped us to substantially improve the quality of our paper. In revising the manuscript, we have carefully considered all the raised comments and suggestions. We have attempted to succinctly explain the changes made in reaction to all comments. Our reply to each comment in a point-by-point fashion is given in what follows.

1. **Regarding your concern about the abstract**

Following your good comments we have corrected the opening sentence of our abstract. We have highlighted it as follows in our manuscript.

*Following the approval of sustainable development goals at the global level, the link between fiscal policy, institutional quality, and economic growth has attracted special attention in economic literature.*

Thank you so much for your comments that improved our paper.

We have also explained the scope of the study in the abstract as follows.

*This study scrutinizes the effect of government revenue-institutional quality interaction on the economic growth of 43 Sub-Saharan African countries for the period of 2012-2022.*

Novelty statement is also included. We have written it as follows.

*The novelty of this study emanates from the estimation technique designated and the introduction of revenue-institutional quality into the economic growth model of SSA. The result of the study reveals*

2. **Regarding your concern about the introduction**

*The introduction is well written however, certain basics are lacking in it. I provided a few as follows; (i) the objective of this study is vague and ambiguous. It should be made explicit and concise. The link between government revenues, institutional quality, and economic growth is not well situated. The authors should improve on it.*

*Dear Reviewers,* thank you for appreciating the introduction section of our manuscript.

As we did it under the abstract, we have written the introduction more clearly (see page 3 paragraph 4).

We have improved the way we wrote the link between government revenue, institutional quality, and economic growth (we have highlighted improved paragraphs in line with your comments).

3. **Regarding your concern regarding the significance of the study**

- Authors should carefully provide relevant contributions of their study to the literature. For instance, how does this study relate to certain SDGs and African Vision 2063? Besides, how your study will improve the current situation in SSA should be clearly stated. Dear reviewers, thank you so much for your comment that improved our paper. We have included the contribution of our paper from the point of view of SDG and Vision 2063 of Africa in the introduction section of our manuscript.

4. **Regarding your concern regarding the theoretical Literature review section**

In line with your good comments, we have summarized Theoretical literature into only four paragraphs (see pages 4 and 5 of the manuscript). We have highlighted the changes that were made to our manuscript. We appreciate your comment that substantially improved our work.

5. **Regarding your concern regarding the Methodological contributions of the study**

In line with your comment, we have explained why the System GMM estimation technique best fits into the analyses (see page 18, section 3.4).

6. **On your concerns Regarding the Empirical Literature review (question 6 to 8 of reviewers comment)**

Following your good comments, we have reduced the empirical review sections to the main variables. We have merged sections and significantly reduced the review page (see sections 2.2 and 2.3 of our revised manuscript or pages 6-12 of the revised manuscript).

7. **Regarding your concern about the model of the study (System GMM model)**

Thank you for recommending Asong Simplice’s work for our reference. We have benefited a lot from his work. We have checked all Equations (1-5) in our model including our variables. We have carefully incorporated your comments.

8. **You do not need a unit root for the model estimated with GMM.**

Thank you so much for your good comment. We are convinced by your comment and eliminated the section of the unit root test. Since the data is short-time data (only eleven years of data), the unit root (random walk of the variables) is not the major threat.

9. **The analysis on the Hausman test is not relevant for your study since system GMM is far better than difference GMM.**

Following your good comments, we have avoided the section of the Hausman test. It is almost a universal fact among economists and other researchers in the area that a two-step system GMM is better and provides a more efficient estimator than difference GMM. Moreover, the fundamental fact that two-step GMM is also more efficient is also another very important fact. Your comments are appreciated.

10. **The discussion of the results should be linked to recent studies.**

In line with your comments, we have linked the result of the study with empirical literature. Thank you so much again for your comments.

11. **The conclusion can be improved.**

We have read and checked our conclusion. Thank you for providing us a chance to edit and recheck the conclusion of our study.

12. **The policy recommendations are weak in their present form.**

Incorporating your comments into the work, we have significantly improved recommendations. We strengthened the policy implication (page 27 paragraphs 2 and 4, page 28, paragraph 1).

13. **Limitations and future research opportunities are missing. They should be considered.**

In line with your comments, we have included future research and limitations of the study (page 28 before reference).

14. **Regarding your concern about typos and trivial grammatical errors**

Following your comments, we have carefully read the work and corrected the grammatical errors.
